# HOMEFOOD randomized trial—beneficial effects of 6-month nutrition therapy on body weight and physical function in older adults at risk for malnutrition after hospital discharge

**DOI:** 10.1038/s41430-022-01195-2

**Published:** 2022-08-26

**Authors:** B. S. Blondal, O. G. Geirsdottir, A. M. Beck, T. I. Halldorsson, P. V. Jonsson, K. Sveinsdottir, A. Ramel

**Affiliations:** 1 Faculty of Food Science and Nutrition, School of Health Science, University of Iceland, Reykjavik, Iceland; 2 University College Copenhagen, Institute of Nursing and Nutrition, Sigurdsgade 26, 2200 Copenhagen, Denmark; 3 The Dietetic and Nutritional Research Unit, EFFECT, Herlev and Gentofte University Hospital, Borgmester Ib Juuls Vej 50, 2730 Herlev, Denmark; 4 The Icelandic Gerontological Research Institute, Tungata 26, 101 Reykjavik, Iceland; 5 Faculty of Medicine, School of Health, University of Iceland, Reykjavík, Iceland; 6 Department of Geriatrics, The National University Hospital of Iceland, Reykjavík, Iceland; 7 Matís ohf, Vinlandsleið 12, 113 Reykjavik, Iceland

**Keywords:** Nutrition, Geriatrics

## Abstract

**Background/objectives:**

Malnutrition is common among older adults. Dietary intervention studies in older adults aiming to improve anthropometrics measures and physical function have been inconsistent. We aimed to investigate the effects of nutrition therapy in combination with home delivered meals and oral nutritional supplements (ONS) in community-dwelling older adults discharged from hospital.

**Methods:**

A total of 106 participants (>65 years) were randomized into the intervention group (*n* = 53) and into the control group (*n* = 53). The intervention group received individual nutrition therapy (five in person visits and three phone calls) and freely delivered energy- and protein- rich foods, while the control group received standard care. Dietary intake, anthropometrics, and short physical performance battery (SPPB) were assessed at baseline and at endpoint.

**Results:**

Energy intake at baseline was similar in both groups (~1500 kcal at the hospital) but there was a significant increase in energy intake and body weight in the intervention group (+919 kcal/day and 1.7 kg, *P* < 0.001 in both cases) during the study period, compared to a significant decrease in both measures among controls (−815 kcal/day and −3.5 kg, *P* < 0.001 in both cases). SPPB score increased significantly in the intervention group while no changes were observed among controls.

**Conclusions:**

Most Icelandic older adults experience substantial weight loss after hospital discharge when receiving current standard care. However, a 6-month multi-component nutrition therapy, provided by a clinical nutritionist in combination with freely delivered supplemental energy- and protein-dense foods has beneficial effects on body weight, physical function, and nutritional status.

**Study registration:**

This study was registered at ClinicalTrials.gov (NCT03995303).

## Introduction

Malnutrition, which is commonly observed among older adults [1, 2], is strongly associated with altered body composition, diminished physical and mental function as well as other adverse clinical outcomes. There is, however, some evidence from observational studies that aging per se is not inevitably associated with malnutrition and that appropriate dietary intake and adequate nutritional status is strongly associated with a reduced risk of mobility limitations and improved quality of life [3–5]. Hospitalizations are usually short as the health care system is overburdened, and if malnutrition is diagnosed in a patient, there might not be enough time to reverse poor nutritional status during the hospital stay. This should shift the emphasis of treatment to the patient’s home after hospital discharge [6].

In older adults discharged from hospital, there are several options which can potentially help to improve dietary intake, e.g., Meals on Wheels (MOW), oral nutritional supplements (ONS) or nutrition therapy provided by a clinical nutritionist/dietitian. According to a recent systematic review [7], MOW interventions in older adults showed significant improved effects on total energy intake and the number of consumed meals/day to be important. Only three studies out of twelve in this review reported outcomes on functional measures, which are more relevant than simple measures of absolute energy intake. In Iceland, standard care among older adults after discharge from hospital is to be able to order MOW, supplying one hot meal a day, but a recent study suggests that such service may be inadequate for frail and sick older adults at nutritional risk [8].

The use of ONS is another way to improve nutritional status but meta-analyses of such interventions have only shown modest benefits with respect to weight gain (~1.0 kg) and improvements in nutritional status [9]. However, there is some suggestion that inclusion of dietary counselling in such interventions might increase efficacy of these two outcomes [8, 10–15].

Very few studies have investigated the combined effects of nutrition therapy and the use of ONS in older adults. Thus, we conducted a 6-month randomized controlled dietary intervention study with the aim of investigating the effects of nutrition therapy provided by a clinical nutritionist following the principles of the Nutrition Care Process (NCP) [16]. This involved access to freely delivered supplemental energy- and protein-dense foods and ONS in community-dwelling older adults discharged from hospital.

## Subjects and methods

### Study design

The HOMEFOOD study was a 6-month, randomized controlled, assessor blinded intervention trial conducted in older adults (age 66–95 years) recruited in the Reykjavik capital area, Iceland between January 2019, and July 2020. The primary aim was to investigate the effects of intense nutritional therapy, including free access to energy- and protein-dense foods delivered to subjects recently discharged from hospital. The primary outcomes of this trial were changes in body weight and physical function (Short Physical Performance Battery (SPPB)). Body weight loss and poor physical function are both important predictors of negative health outcomes in older adults [2, 17]. These two variables were chosen to be the primary outcomes, as a nutrition intervention with focus on increasing energy- and protein intake is likely to affect body weight and physical function [4, 18], considering the low energy intake previously reported in elderly discharged patients [19]. Secondary outcomes included other anthropometric measurements, nutritional status, muscular strength, dietary intake, exercise, and reported food-related digestion issues, such as diarrhoea, nausea, constipation, or stomach pain.

### Reporting, approval, and funding

This study was conducted and reported according to the Consolidated Standards of Reporting Trials guidelines for Randomized Trials of Nonpharmacologic Treatments (CONSORT) [20]. The study was approved by the Ethics Committee for Health Research of the National University Hospital of Iceland and data protection registry (24/2018) in August 2018 and performed in accordance with the Declaration of Helsinki [21]. The study was registered at ClinicalTrials.gov (NCT03995303).

### Screening and recruitment

**Fig. 1:**
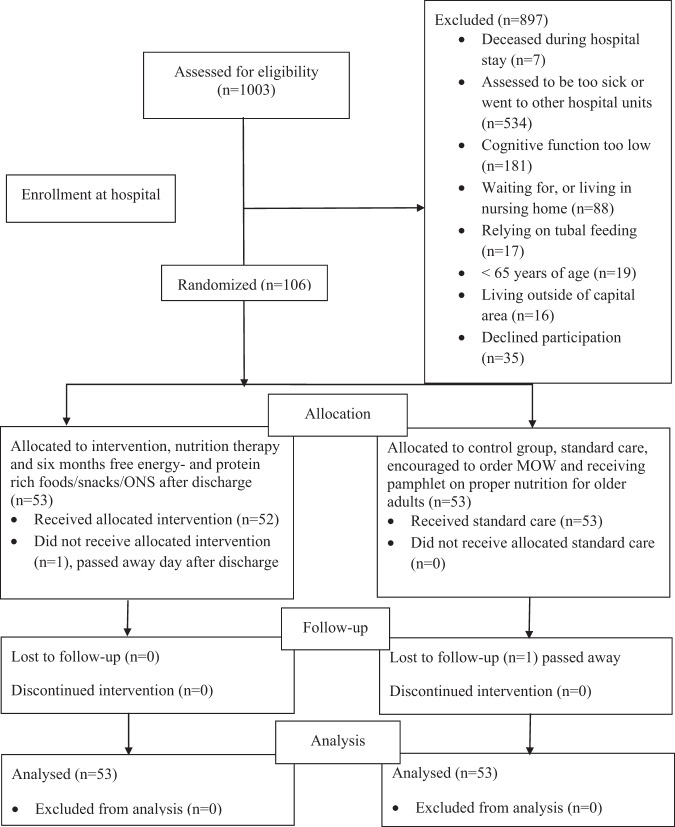
Flow chart. Flow chart of assessment, recruitment, allocation, follow up, and analysis process.

Potential participants (*N* = 1003) were screened by a clinical nutritionist in collaboration with attending nurses at the Landspitalinn University Hospital of Iceland. Eligible patients were discharging home to independent living from the hospital, aged 65 years or older, and assessed as being at risk for malnutrition (score ≥ 3) according to the validated Icelandic Nutrition Screening Tool [22], and had given their written informed consent. Excluded were those with known dietary allergies/being on a special diet, severe chronic kidney disease (glomerular filtration rate < 30 mL/min/1.73 m^2^), in active cancer treatment, receiving tubal feeding, not being able to communicate with the research team, cognitive function ≤20 according to the Mini Mental State Examination (MMSE) [23], and not having access to a functioning kitchen at home (i.e., refrigerator, oven, or microwave oven). Of the 1003 screened potential participants, *n* = 897 were ineligible for participation in the study. They were ineligible as they were too sick to participate, had been discharged, deceased, scored <20 on the MMSE, had been admitted to a nursing home, were not community dwelling, relying on tubal feeding, were <65 years of age, were not living in the capital area, or had declined participation (Fig. 1).

### Randomization

The participants were randomly allocated to either the intervention or the control group by using a random number generated as implemented by the Statistical Package for the Social Sciences (SPSS, version 26.0, SPSS, Chicago, IL, USA).

### Intervention group

The participant received nutrition therapy from the clinical nutritionist consisting of five home visits (1 day after discharge and one-, three-, six- and twelve weeks later) and three telephone calls in between the home visits. The nutrition therapy was implemented following the principles of Nutrition Care Process, which entails the following: nutritional assessment, diagnosis, intervention, monitoring, and evaluation of the nutrition therapy [16]. During the dietary counselling sessions, family members, relatives, friends, or home-care workers were invited to join as well. At the initial visit after discharge, the participant was educated about the importance of adequate energy and protein intake [24]. Nutrition-related problems were identified during the interviews, and suggestions given to resolve them. In addition to the dietetic counselling, participants received free supplemental energy- and protein-rich foods (1 hot meal/day and 2 in-between-meals/day; Supplementary Table 1) delivered once a week. During the first home delivery, study staff educated the participants on how to store the meals, how to open the packages and how to heat the meals.

### Control group

At discharge, the control group received a booklet on good nutrition during aging published by The Icelandic Medical Directorate [24] and were encouraged to order MOW without any further dietary counselling during the study period, reflecting current standard care in Iceland for older adults discharged from hospital.

### Participant characteristics

Background variables, e.g., age, sex, education, living arrangements, alcohol use and smoking habits, were assessed using questionnaires. Additional variables were collected from the Icelandic electronic hospital registry SAGA (TM software 3.1.39.9), e.g., height, number of diagnoses according to the International Statistical Classification of Diseases and Related Health Problems 10th Revision (ICD-10), and number of different medications.

### Outcomes assessed

All primary and secondary outcome measurements were conducted at baseline (at the hospital) and at endpoint (at the participants’ homes). These measurements were conducted in a predefined order and questions on food or diet were asked only at the very end of each assessment. As the outcome assessors (who did not deliver the intervention) were unaware whether a participant was in the control or intervention group, measurements of anthropometrics, physical function, muscular strength, and nutritional status were blinded.

#### Anthropometrics

Body weight was measured in light underwear/clothing on a calibrated scale (model no. 708, Seca, Hamburg, Germany) and height was taken from the Icelandic electronic hospital registry SAGA (TM software 3.1.39.9). Body mass index (BMI) was calculated from the height and weight (kg/m^2^). Participants were categorized into three BMI categories: low BMI < 23 kg/m^2^, middle BMI 23–30 kg/m^2^, or high BMI ≥ 30 kg/m^2^ [24]. Body composition was measured using a hand-held bioelectrical impedance analysis device (BIA, Omron HBF-306C, Kyoto, Japan) [25]. Calf circumference was measured in a seated position. The tape was wrapped around the right calf and moved up and down to locate the maximum circumference in a plane perpendicular to the long axis of the calf [26]. Midarm circumference was also measured in a seated position and was taken on the left upper arm, at the mid-point between the tip of the shoulder and the tip of the elbow (olecranon process and the acromion) [27].

#### Physical function

Physical function was assessed using the SPPB, which evaluates lower extremity function assessing (1) usual-paced gait speed over a four-meter-course, (2) standing balance, and (3) time to rise from a chair five times. For each test, a score of 0 to 4 is assigned using cut points [28]. The three test scores are summed, yielding a range from 0 to 12. As SPPB testing in this study was performed at the participants’ homes, it was shortened for practical reasons, and thus did not include the gait speed part. The possible score therefore ranged from 0 to 8. Additionally, participants were asked the question “Do you have difficulties walking?” (Yes vs. no).

#### Muscle strength

Handgrip strength was measured in a seated position with a hydraulic hand dynamometer (Baseline^®^ Baseline Evaluations Corporation) set on position two and the maximal grip strength of two trials was registered as the subject’s grip force in kilograms using their dominant hand [27].

### Dietary intake

Dietary intake was assessed using a 24-hour-dietary-recall interview (24-HR) to obtain estimates of intakes of fluids, energy, and energy-giving nutrients [29–34]. The results from the 24-HR were entered into the nutrition calculation program ICEFOOD originally developed for the National Survey of Icelandic Diet 2002 and continuously updated for consequent National Surveys of Icelandic Diet (2011 and currently ongoing) [35]. ICEFOOD relies on the Icelandic database of the chemical composition of food (ISGEM within the Icelandic Medical Directorate of Health) and on a database within the Medical Directorate containing information on several hundred recipes of common dishes and ready-to-eat meals on the Icelandic market [35, 36]. Additional food-related questions and frequency of intakes of hot meals, major food groups, and liquids were assessed at endpoint using a simple food frequency questionnaire [37].

#### Nutritional status and food-related adverse events

Nutritional status was assessed using the Icelandic Nutrition Screening Tool as recommended by the Icelandic Medical Directorate of Health [24]. This validated questionnaire [22] consists of 13 questions which are scored and summed, yielding a range from 0 to 30. A clinical nutritionist also assessed whether any food- related digestion issues, such as diarrhoea, nausea, constipation, or stomach pain, were experienced during the intervention.

### Sample size considerations

Sample size calculations based on our previous studies on body weight change [19, 38] suggest that the number of participants *n* = 44 in each group was estimated to be sufficient to detect a body weight difference of 1.8 ± 3.0 kg between groups as significant. The corresponding numbers for SPPB were *n* = 45 in each group, detecting a significance by 1 as significant (assuming SD = 1.7) [39]. The recruitment of >50 participants in each group allowed around 10% drop out to still retain sufficient statistical power.

### Statistical analysis

Data were analysed using statistical software (SPSS, version 26.0, SPSS, Chicago, IL, USA). Data were checked for normality using the Kolmogorov–Smirnov test. Data are presented as mean ± standard deviation (SD). Differences between groups at baseline were calculated using independent samples’ *t*-test (normally distributed variables) or Mann–Whitney *U*-test (not normally distributed variables). We used intention-to-treat analysis.

Despite randomization, sex distribution was slightly uneven between treatment and control groups. As a result, we corrected for sex in all multivariate statistical endpoint analyses. Unadjusted analyses are also presented for comparison as supplemental material (Supplementary Table 2). Differences in anthropometrics and physical outcomes (continuous variables) between the groups at endpoint were assessed using linear mixed models in SPSS. Results are shown as parameter estimates, in which B describes the estimated and adjusted differences in the outcome variables between groups.

Differences in the abilities to perform physical tasks (single items from SPPB and “Do you have difficulties walking?” all categorical variables, yes vs. no) between the groups at endpoint were assessed using a logistic regression model, in which we corrected for the corresponding baseline values and sex.

Subgroup analysis was performed by comparing body weight changes between intervention and control in subgroups of males vs. females, married/cohabitating vs. single/divorced/alone, and low BMI group vs. middle BMI group vs. high BMI group. The effects of the intervention within subgroups were investigated using an independent samples’ *t*-test (for two variable subgroups) or ANOVA including LSD post hoc test (for three variable subgroups). We tested for interaction between subgroups and intervention using a general linear model.

Endpoint calculations represent per-protocol analysis with those dropping out of the intervention included in the baseline, but not in endpoint assessment. The level of significance was set at *P* < 0.05.

## Results

During the recruitment period, 1003 subjects were screened and of those 106 were recruited and randomized. Two subjects dropped out during the study period, one from each group (Fig. 1). The study was carried out as planned and all participants in the intervention group (with exception of the one dropout) received five home visits and three phone calls. No discomfort or adverse events relating to the intervention were observed among study participants.

Baseline characteristics of the participants are shown in Table 1. The intervention and control groups were similar in most measures, with the exception that there were significantly more females in the intervention group compared to controls (72 vs 53%). In agreement with this uneven sex distribution, the intervention group also had a higher body fat percentage (borderline significant).

**Table 1:** Characteristics of the participants.

Variables	Control	%	Intervention	%	*P*-value^a^
	(*n* = 53)		(*n* = 53)		
	Mean ± SD		Mean ± SD		
Age (years)	81.8 ± 6.0		83.3 ± 6.7		0.228
Female		52.8		71.7	0.045
Higher education (yes)		66.0		69.8	0.677
Lives alone (yes)		62.3		66	0.685
Alcohol (yes)		45.3		37.7	0.43
Smoking (yes)		9.4		3.8	0.241
ISNST score	4.5 ± 1.3		5.1 ± 1.7		0.047
MMSE score	25.9 ± 2.9		26.1 ± 2.8		0.702
No. of ICD-10 diagnoses	10.5 ± 3.8		10.3 ± 4.9		0.877
No. of medications	12.4 ± 4.2		12.2 ± 5.8		0.893
Height (m)	1.7 ± 0.1		1.7 ± 0.1		0.326
Weight (kg)	76.5 ± 19.1		78.3 ± 18.3		0.615
BMI (kg/m^2^)	26.9 ± 5.3		28.5 ± 6.5		0.188
Waist circumference (cm)	104.4 ± 14.0		103.6 ± 13.8		0.739
Mid arm circumference (cm)	28.3 ± 4.0		29.8 ± 5.7		0.114
Calf circumference (cm)	34.0 ± 4.5		34.9 ± 4.9		0.349
Fat free mass (kg)	49.1 ± 11.9		48.1 ± 10.2		0.629
Fat percent (%)	35.2 ± 8.3		38.3 ± 9.6		0.082
Handgrip strength (kg)	21.5 ± 8.5		19.7 ± 6.8		0.119
SPPB (score)	2.4 ± 2.0		2.5 ± 1.8		0.839

*BMI* body mass index, *ICD-10* International Statistical Classification of Diseases and Related Health Problems 10th Revision, *ISNST* Icelandic Nutrition Screening Tool, *MMSE* Mini Mental State examination, *SPPB* short physical performance battery.

^a^*P*-value based on chi square test for categorical variables, independent samples *t*-test for normally distributed continuous variables and Mann–Whitney *U* test for not normally distributed continuous variables.

**Fig. 2:**
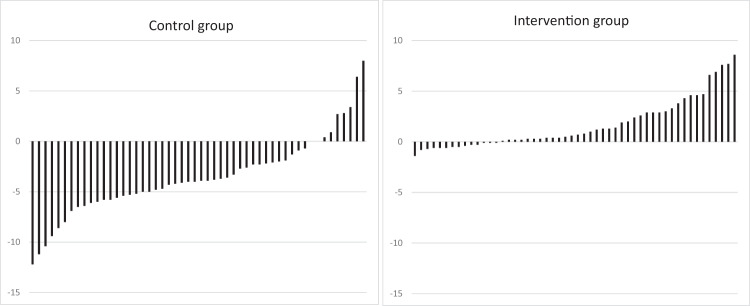
Individual weight changes. Individual crude body weight changes (in kg) 6 months after discharge in the control group and in the intervention group.

Concerning the primary outcome, individual changes in body weight for participants in both groups are shown in Fig. 2. The intervention group experienced in absolute terms significant weight gain during the intervention period (1.7 kg ± 2.5 kg; which equals approximately 2% of body weight, 1 out of 53 individuals lost >1 kg body weight), while significant weight loss was observed among controls (−3.5 ± 3.9 kg; which equals approximately 5% of body weight, 42 out of 53 individuals lost >1 kg body weight). After adjustment for sex (Table 2) this corresponded to 5.1 kg (95% CI: 3.9, 6.4) higher body weight in the intervention group at endpoint compared to controls. The corresponding adjusted difference in lean body mass was 4.2 kg (95% CI: 2.7, 5.6). For other anthropometric outcomes, i.e., BMI, waist-, midarm- and calf circumference, the sex adjusted differences between groups showed significantly lower values in the control group (Table 2).

**Table 2:** Sex adjusted differences in anthropometrics and physical outcomes between the groups at endpoint^a^.

Outcome variable at endpoint	Groups	B	95% CI	*P*-value
Body weight (kg)	control vs. intervention	−5.121	(−6.381, −3.860)	<0.001
Body mass index (kg/m^2^)	control vs. intervention	−1.693	(−2.167, −1.220)	<0.001
Waist circumference (cm)	control vs. intervention	−2.624	(−4.506, −0.743)	0.007
Mid arm circumference (cm)	control vs. intervention	−2.185	(−3.212, −1.158)	<0.001
Calf circumference (cm)	control vs. intervention	−1.266	(−2.33, −0.190)	0.020
Body fat (%)	control vs. intervention	1.260	(−0.575, 3.096)	0.176
Lean body mass (kg)	control vs. intervention	−4.181	(−5.647, −2.715)	<0.001
Hand grip strength (kg)	control vs. intervention	−0.871	(−3.124, 1.155)	0.401
SPPB (score)	control vs. intervention	−0.906	(−1.787, −0.293)	0.024
ISNST score)	control vs. intervention	2.226	(1.381, 3.071)	<0.001

*ISNST* Icelandic Nutrition Screening Tool, *SPPB* short physical performance battery.

^a^Based on linear mixed model adjusted for sex.

As expected, the highest increase in body weight was observed in participants in the low or middle BMI categories (Table 3). The unadjusted means for all anthropometric measures at baseline and endpoint are also shown in Supplementary Table 2.

**Table 3:** Sex adjusted subgroup analysis of the main treatment effect (weight change during study period).

	Control		Intervention^a^		
		mean ± SD		mean ± SD	*P*-value^b^
All (*n* = 106)	*n* = 53	−3.46 ± 3.92	*n* = 53	1.69 ± 2.46	<0.001
Female (*n* = 66)	*n* = 28	−2.64 ± 4.32^a^	*n* = 38	1.58 ± 2.51	<0.001
Male (*n* = 40)	*n* = 25	−4.38 ± 3.27	*n* = 15	1.85 ± 2.41	<0.001
Married/cohabitation (*n* = 38)	*n* = 20	−4.14 ± 3.81^a^	*n* = 18	1.04 ± 1.90	<0.001
Single/divorced/alone (*n* = 78)	*n* = 33	−3.06 ± 3.99	*n* = 35	1.97 ± 2.67	<0.001
Low BMI category (*n* = 21)	*n* = 12	−2.19 ± 3.71^c^	*n* = 9	4.42 ± 2.76	<0.001
Middle BMI category (*n* = 59)	*n* = 30	−4.42 ± 3.97	*n* = 29	1.43 ± 2.16	<0.001
High BMI category (*n* = 26)	*n* = 11	−2.25 ± 3.57	*n* = 15	0.43 ± 1.47	0.036
Age tertile 1 (*n* = 36)	*n* = 21	−3.21 ± 4.34^a^	*n* = 15	1.66 ± 2.89	<0.001
Age tertile 2 (*n* = 40)	*n* = 19	−3.15 ± 4.28	*n* = 21	1.69 ± 2.39	<0.001
Age tertile 3 (*n* = 30)	*n* = 13	−4.34 ± 2.58	*n* = 17	1.62 ± 2.29	<0.001

^a^no significant differences in the treatment effects between the subgroups, e.g., no difference between men and women (reads vertically).

^b^*P*-value is based on an independent samples t-test for the difference between control and intervention (reads horizontally)

^c^Significant differences in the treatment effects between the subgroups according to ANOVA including LSD post hoc test: weight gain (Low BMI) > weight gain (Middle BMI) = weight gain (High BMI). Interaction term in linear analysis *P* = 0.027.

With respect to measures of physical function, the SPPB score was significantly higher in the intervention group at endpoint (Table 2) and subjects in the intervention group were also more likely to improve in single physical performance tasks at endpoint (adjusted results in Table 4 and unadjusted results in Supplementary Table 2). Handgrip strength did not differ statistically between the groups at endpoint (Table 2).

**Table 4:** Likelihood of improvement in performing physical tasks at endpoint adjusted for sex^a^.

Outcome variable^b^	Groups	OR (95% CI)	*P*-value
Being able to perform “side-by-side”	intervention group (*n* = 53)	3.15 (0.98, 10.10)	
	control group (*n* = 53)	1	0.052
Being able to perform “semi-tandem”	intervention group (*n* = 53)	3.77 (1.45, 9.80)	
	control group (*n* = 53)	1	0.007
Being able to perform “tandem”	intervention group (*n* = 53)	2.21 (0.95, 5.10)	
	control group (*n* = 53)	1	0.065
Being able to perform “chair test”	intervention group (*n* = 532)	2.02 (0.80, 8.55)	
	control group (*n* = 53)	1	0.137
Having difficulties walking – yes^c^	intervention group (*n* = 53)	0.34 (0.14, 0.83)	
	control group (*n* = 53)	1	0.018

^a^Based on logistic regression. Adjusted for baseline values and sex.

^b^Physical tasks are single items from the Short Physical Performance Battery as well as the single question “Do you have difficulties walking?”

^c^Having difficulties to walk is not an improvement but a deterioration.

In terms of nutritional status, the Icelandic Nutrition Screening Tool score at endpoint was significantly higher (higher score corresponds to a worse nutritional status) among controls compared to the intervention group (Table 2). No difference in dietary intake between the two groups were detected at baseline (i.e., at the hospital) but energy and macronutrient intake increased significantly in the intervention group and decreased significantly in the control group during the intervention period (Table 5). In the intervention group, ONS provided 24 and 29% of the total energy and protein at endpoint (which equals approximately 1.75 ONS servings/day), respectively. At endpoint, more than 94% of the intervention group also stated that they liked the provided food, reported a higher frequency of hot meals consumed, more frequent consumption of meat, and a higher intake of liquids compared to controls. No food related digestion issues, such as diarrhoea, nausea, constipation, or stomach pain were reported.

**Table 5:** Dietary intake of the participants (baseline, endpoint), food related questions and food frequencies (endpoint).

Variables		Control		Intervention		*P*-value^a^
		(*n* = 53)		(*n* = 53)		
		Mean ± SD		Mean ± SD		
Energy intake (kcal)	baseline	1546 ± 297		1493 ± 360		0.412
	endpoint	731 ± 320		2412 ± 403		<0.001
Protein (g)	baseline	77.3 ± 14.8		74.7 ± 18.0		0.411
	endpoint	31.2 ± 15.5		118.2 ± 34.3		<0.001
Protein (g/kg BW^b^)	baseline	1.1 ± 0.4		1.0 ± 0.3		0.202
	endpoint	0.4 ± 0.2		1.5 ± 0.4		<0.001
Carbohydrates (g)	baseline	135.3 ± 26.0		130.7 ± 31.5		0.411
	endpoint	77.2 ± 34.4		203.5 ± 43.0		<0.001
Fat (g)	baseline	77.3 ± 14.8		74.7 ± 18.1		0.412
	endpoint	31.1 ± 18.3		122.0 ± 29.8		<0.001
Dietary fibre (g)	baseline	22.7 ± 4.4		21.9 ± 5.3		0.413
	endpoint	6.4 ± 4.2		11.2 ± 3.8		<0.001
Do you enjoy food (endpoint)?	yes		73.6%		86.8%	0.088
Do you like the food that you get (endpoint)?	yes		77.4%		94.3%	0.012
How often do you eat a hot meal (endpoint)?	once or twice a week		11.3%		0.0%	0.003
	3-4 times a week		7.5%		0.0%	
	5-6 times a week		11.3%		1.9%	
	every day		67.9%		96.2%	
	more than once a day		1.9%		1.9%	
How often do you eat meat (endpoint)?	less than once a week		3.8%		0.0%	0.007
	once or twice a week		24.5%		3.8%	
	3-4 times a week		67.9%		88.7%	
	5-6 times a week		3.8%		7.5%	
How often do you eat vegetables (endpoint)?	never		3.8%		0.0%	0.101
	less than once a week		7.5%		3.8%	
	once or twice a week		22.6%		9.4%	
	3-4 times a week		26.4%		37.7%	
	5-6 times a week		7.5%		18.9%	
	every day		32.1%		30.2%	
How often do you eat fish (endpoint)?	less than once a week		7.5%		0.0%	0.096
	once or twice a week		22.6%		13.2%	
	3-4 times a week		66.0%		83.0%	
	5-6 times a week		3.8%		3.8%	
How much liquid do you drink (endpoint)?	one to two cups a day		3.8%		0.0%	0.014
	3-4 cups a day		18.9%		1.9%	
	5-6 cups a day		52.8%		66.0%	
	7 or more cups a day		24.5%		32.1%	
How much butter do you use on bread (endpoint)?	little butter		9.4%		11.3%	0.093
	medium butter		60.4%		39.6%	
	thick butter		30.2%		49.1%	

^a^*P*-value for the differences between groups. Based on independent samples *t*-test for continuous variables and based on chi-square statistics for categorical variables.

^b^g/kg BW = daily protein intake in g/kg body weight.

In terms of stability analyses our analyses indicated (Table 3) that neither sex, marital status, nor participant age affected the efficacy of the intervention. However, body weight changes differed by BMI categories and interaction between BMI categories and intervention was significant (*P* = 0.027).

## Discussion

In this 6-month randomized, controlled intervention we examined the effects of nutrition therapy provided by a clinical nutritionist following the principles of NCP [16] in combination with freely delivered supplemental energy- and protein-dense foods in older adults after discharge from hospital. We found that this nutrition intervention had strong beneficial effects on body weight and other anthropometric measures; as well as SPPB score, and nutritional status. The observed effects in our study were more pronounced than what has been reported in previous nutritional interventions not combining ONS, MOW, and nutrition therapy from a nutritionist.

Changes in body weight were observed in both groups, with an average of 5.1 kg higher body weight (which equals roughly a 7% difference in body weight) among those receiving the intervention, who gained a moderate amount of weight, compared to controls, who lost weight, which agrees with changes in dietary intake recordings after hospital discharge. The fact that 42 out of 53 of participants in the control group lost more than 1 kg body weight while only one individual in the intervention lost that much weight demonstrates that individual and targeted nutrition therapy in combination with the provision of ONS and MOW, can largely prevent negative alterations in body weight after hospital discharge.

There are currently no studies available in public literature that use the combination of nutrition therapy, home delivered food, and ONS in older adults which would allow direct comparison of the results. However, two recent trials using three home visits by a registered dietitian as intervention showed significant body weight gain in discharged patients, resulting in significant endpoint differences of 1.4–1.8 kg (~2–3% of body weight) between intervention and control [14, 15], although these studies were shorter in length (12 weeks) and did not deliver food items. Recent review articles on the efficacy of ONS, MOW and dietary advice [9, 37, 40, 41] to increase dietary intake and body weight yielded results in the range of 200–400 kcal/d and 0.6–1.5 kg (~1.5-2% of body weight), respectively. Although significant, the effect sizes were small and possibly, long-term compliance to, e.g., ONS or MOW, decreases over time and ONS might displace food rather than serve as an addition to regular dietary intake in the long run [42, 43].

Although it is difficult to accurately estimate body composition during a home visit, we employed various methods (hand-held BIA, upper arm- and calf circumference) to get insight into changes in lean body mass during the intervention. Results from the various measurements were consistent and data from BIA showed that weight loss in the control group was mainly due to loss of lean body mass, and weight gain in the intervention group was mainly due to an increase in lean body mass (and not body fat).

In the present study the nutrition intervention also had favourable effects on physical function, although we could not detect any changes in muscular strength. We found that both objectively measured physical function (SPPB) as well as subjectively experienced difficulties in walking improved only in the intervention group during the 6 months. In general, the evidence on the effects of nutrition intervention on physical function and muscular strength in older adults is limited. Although several studies using either MOW or ONS found effects on physical function [44, 45] and muscular strength [44], other studies did not (physical function: [46], muscular strength: [45, 47–49]).

In the present study, dietary intake at baseline was similar between groups at around 1500 kcal and 75 g protein per day which reflects the food provided by the hospital. Similar numbers have been previously reported by other investigators as well [50]. However, after discharge, the dietary intake decreased dramatically in the control group despite being informed at discharge of the importance of nutrition, while intake increased considerably in the intervention group. Interestingly, a low dietary intake nearly identical to the control group’s intake was observed in a small pilot study in discharged hospital patients conducted by our research group in 2016 [19].

The results from our study are of potential public health importance for the following reasons: It is known that malnutrition is common in hospitalized older adults [1, 2, 51, 52] and unfortunately, our study demonstrates that weight loss continues after discharge in most participants who receive standard care, which has also been observed to some extent by other researchers [53]. Further, the treatment effects of our intervention, utilizing three components to improve nutritional status, were higher than reported from other studies having used only single modalities of nutrition intervention, e.g., ONS, MOW, or dietary advice [9, 40, 54–57]. It seems obvious that the wider approach of our nutrition therapy based on principles of NCP, in combination with the delivery of a variety of foods that were highly energetic and rich in protein while leaving space for individual needs and personal preferences, resulted in high acceptance of the delivered foods even after 6 months, satisfactory dietary intake, and body weight gain.

The current intervention was successful in improving body weight and maintaining muscle mass in discharged patients. The subgroup analysis indicated that the treatment effects were similar between subgroups, except in the three BMI categories, where participants in the high BMI category gained less body weight than participants in the middle and low BMI categories, which simply reflects different aims of the nutrition therapy dependent on individual characteristics of the participants. In our opinion the success of our study can be attributed to (1) the individualized and frequent nutritional therapy performed by a dedicated clinical nutritionist, (2) the provision of food developed to be palatable for older adults, rich in energy and protein as well as with the appropriate texture; and (3) the length of the intervention being 6 months leading to significant improvements in both nutritional status and physical function.

### Strengths and limitations

It is a strength of the present study that it was a randomized, controlled trial with very low drop out and 100% delivery of the intended intervention in 52 of 53 participants. Although a study like this cannot be doubly blinded, it is of importance that the assessment of the main outcomes was single blinded. We think that both the time length (6 months) and the intensity of intervention (five visits, three phone calls and free home delivered food) were appropriate to be able to observe potential treatment effects.

There are several limitations to our study. One is the gender imbalance between the control and the intervention group. Despite this gender imbalance our adjusted and unadjusted outcomes reach the same conclusion; nearly all subjects in the control group lost weight while almost all in the intervention group gained or maintained their weight, which cannot be explained by sex alone. Also, with our sample size being 106, some imbalances in the baseline factors can be expected and the method used for randomization was valid. Another limitation was that as outcome measurements were conducted at the patients’ homes, we were limited in the assessment of physical function, i.e., lacking a measurement of gait speed, as well as in the measurement of body composition, i.e., having to rely on hand-held BIA and circumference measurements. However, we still collected valuable information about both body composition and physical function from which we can draw solid conclusions.

## Conclusion

Our study shows that the time after hospital discharge leads to weight loss and loss of muscle mass, a decrease in food intake, and a deterioration in nutritional status in most older adults receiving the current standard care in Iceland. However, a 6-month nutrition therapy provided by a clinical nutritionist, following the principles of NCP in combination with freely delivered supplemental energy- and protein-dense foods, has beneficial effects on body weight, physical function, dietary intake, and nutritional status. The treatment effects were consistent across subgroups of study participants.

## Supplementary information

Supplemental material

## Data Availability

Data described in the manuscript, code book, and analytic code are publicly and freely available without restriction at: [https://eur02.safelinks.protection.outlook.com/?url=https%3A%2F%2Fdataverse.harvard.edu%2Fdataset.xhtml%3FpersistentId%3Ddoi%3A10.7910%2FDVN%2F38X3LX&data=04%7C01%7Cbsb6%40hi.is%7C1e998a8333904a1087a008d9803f56dd%7C09fa5f0e211846568529677ed8fdbe78%7C0%7C0%7C637681831802708546%7CUnknown%7CTWFpbGZsb3d8eyJWIjoiMC4wLjAwMDAiLCJQIjoiV2luMzIiLCJBTiI6Ik1haWwiLCJXVCI6Mn0%3D%7C1000&sdata=LJU6Bm%2F9up1VO8rsWUdMnf5d5WewkRQpRUzfpmr8GgM%3D&reserved=0].
