# Supplementary material for: HOMEFOOD randomized trial—beneficial effects of 6-month nutrition therapy on body weight and physical function in older adults at risk for malnutrition after hospital discharge
Source: Eur J Clin Nutr. 2022 Aug 26;77(1):45–54. doi: 10.1038/s41430-022-01195-2 (PMC9876791; doi:10.1038/s41430-022-01195-2)
Supplement: Supplementary file 1 — Supplemental material [file 41430_2022_1195_MOESM1_ESM.docx]

**Supplemental table 1** Supplemental food items

|  |  |  | **Energy** | **protein** | **portion size** | **energy** | **protein** |
| --- | --- | --- | --- | --- | --- | --- | --- |
| **Food item categories** | **producer** | **name** | **kcal/100 g** | **g/100 g** | **(g)** | **kcal/portion** | **protein/portion** |
| Meals | Slaturfelag Suðurlands Ltd. | Meat balls | 109 | 8.5 | 450 | 491 | 38.3 |
|  |  | Lamb in Béarnaise sauce | 143 | 9.1 | 450 | 644 | 41 |
|  |  | Lamb sausage | 146 | 6 | 450 | 657 | 27 |
|  |  | Meat loaf | 114 | 6 | 450 | 513 | 27 |
|  |  | Cabbage meat rolls | 121 | 4.5 | 450 | 545 | 20.3 |
|  |  | Rice-liver-pudding | 164 | 6.4 | 360 | 590 | 23.2 |
|  |  |  |  |  |  |  |  |
|  | Grimur kokkur Ltd. | Salted cod | 156 | 8.5 | 450 | 702 | 38.3 |
|  |  | Fish stew | 143 | 8.5 | 450 | 644 | 38.3 |
|  |  | Fish balls | 128 | 8.5 | 450 | 576 | 38.3 |
|  |  | Fish burger | 133 | 8.5 | 450 | 599 | 38.3 |
|  |  |  |  |  |  |  |  |
| In between meals | MS Iceland Dairies | SMS curd | 61 | 4.9 | 100 | 61 | 4.9 |
|  |  |  |  |  |  |  |  |
| ONS | MS Iceland Dairies | Nutrition + (chocolate) | 132 | 8.1 | 250 | 330 | 20.3 |
|  |  | Nutrition + (coffee) | 132 | 8 | 250 | 330 | 20 |

ONS = Oral nutrition supplements

**Supplemental table 2** Unadjusted baseline and endpoint values in the control- and intervention- group.

|  |  | **Control group** | |  | **Intervention group** | |
| --- | --- | --- | --- | --- | --- | --- |
|  |  | **(n = 53)** |  |  | **(n = 53)** |  |
|  |  | **Mean ± SD** |  |  | **Mean ± SD** |  |
| Body weight (kg) | baseline | 76.5 ± 19.2 |  |  | 78.3 ± 18.2 |  |
|  | endpoint | 73.0 ± 19.3 |  |  | 80.0 ± 17.4 |  |
| Body mass index (kg/m^2^) | baseline | 26.9 ± 5.3 |  |  | 28.5 ± 6.5 |  |
|  | endpoint | 25.8 ± 5.5 |  |  | 29.0 ± 6.1 |  |
| Waist circumference (cm) | baseline | 104.3 ± 13.9 |  |  | 103.6 ± 13.8 |  |
|  | endpoint | 101.9 ± 14.2 |  |  | 103.8 ± 13.5 |  |
| Upper arm circumference (cm) | baseline | 28.3 ± 4.0 |  |  | 29.8 ± 5.7 |  |
|  | endpoint | 27.5 ± 4.9 |  |  | 31.2 ± 5.9 |  |
| Calf circumference (cm) | baseline | 34.0 ± 4.5 |  |  | 34.9 ± 4.9 |  |
|  | endpoint | 33.7 ± 5.8 |  |  | 35.9 ± 4.5 |  |
| Body fat (%) | baseline | 35.3 ± 8.5 |  |  | 37.7 ± 9.0 |  |
|  | endpoint | 36.8 ± 7.6 |  |  | 37.9 ± 8.3 |  |
| Lean body mass (kg) | baseline | 49.0 ± 11.7 |  |  | 48.0 ± 10.3 |  |
|  | endpoint | 45.8 ± 11.69 |  |  | 49.1 ± 10.4 |  |
| Grip strength (kg) | baseline | 21.5 ± 8.3 |  |  | 19.7 ± 6.8 |  |
|  | endpoint | 21.3 ± 9.3 |  |  | 20.4 ± 8.9 |  |
| SPPB (score) | baseline | 2.4 ± 1.9 |  |  | 2.5 ± 1.8 |  |
|  | endpoint | 2.8 ± 2.2 |  |  | 3.8 ± 2.0 |  |
| ISNST (score) | baseline | 4.5 ± 1.3 |  |  | 5.1 ± 1.7 |  |
|  | endpoint | 3.7 ± 2.2 |  |  | 2.0 ± 1.3 |  |
|  |  |  |  |  |  |  |
|  |  |  | **yes in %** |  |  | **yes in %** |
| Being able to perform "side-by-side" | baseline |  | 71.7 |  |  | 77.4 |
|  | endpoint |  | 75.5 |  |  | 90.6 |
| Being able to perform "semi-tandem" | baseline |  | 67.9 |  |  | 66.0 |
|  | endpoint |  | 58.5 |  |  | 83.0 |
| Being able to perform "tandem" | baseline |  | 37.7 |  |  | 41.5 |
|  | endpoint |  | 49.1 |  |  | 67.9 |
| Being able to perform "chair test" | baseline |  | 81.1 |  |  | 86.8 |
|  | endpoint |  | 66.0 |  |  | 81.1 |
| Having difficulties to walk | baseline |  | 28.3 |  |  | 34.0 |
|  | endpoint |  | 45.3 |  |  | 24.5 |

ISNST = Icelandic Nutrition Screening Tool, SPPB = short physical performance battery.
